# Supplementary material for: TGF-β signaling modulates adhesion and actomyosin contractility to pattern epithelial permeability in Drosophila ovaries
Source: J Cell Sci. 2026 Oct 12;139(21):jcs264997. doi: 10.1242/jcs.264997 (PMC13741950; doi:10.1242/jcs.264997)
Supplement: Supplementary file 1 [file joces-139-264997-s1.pdf]

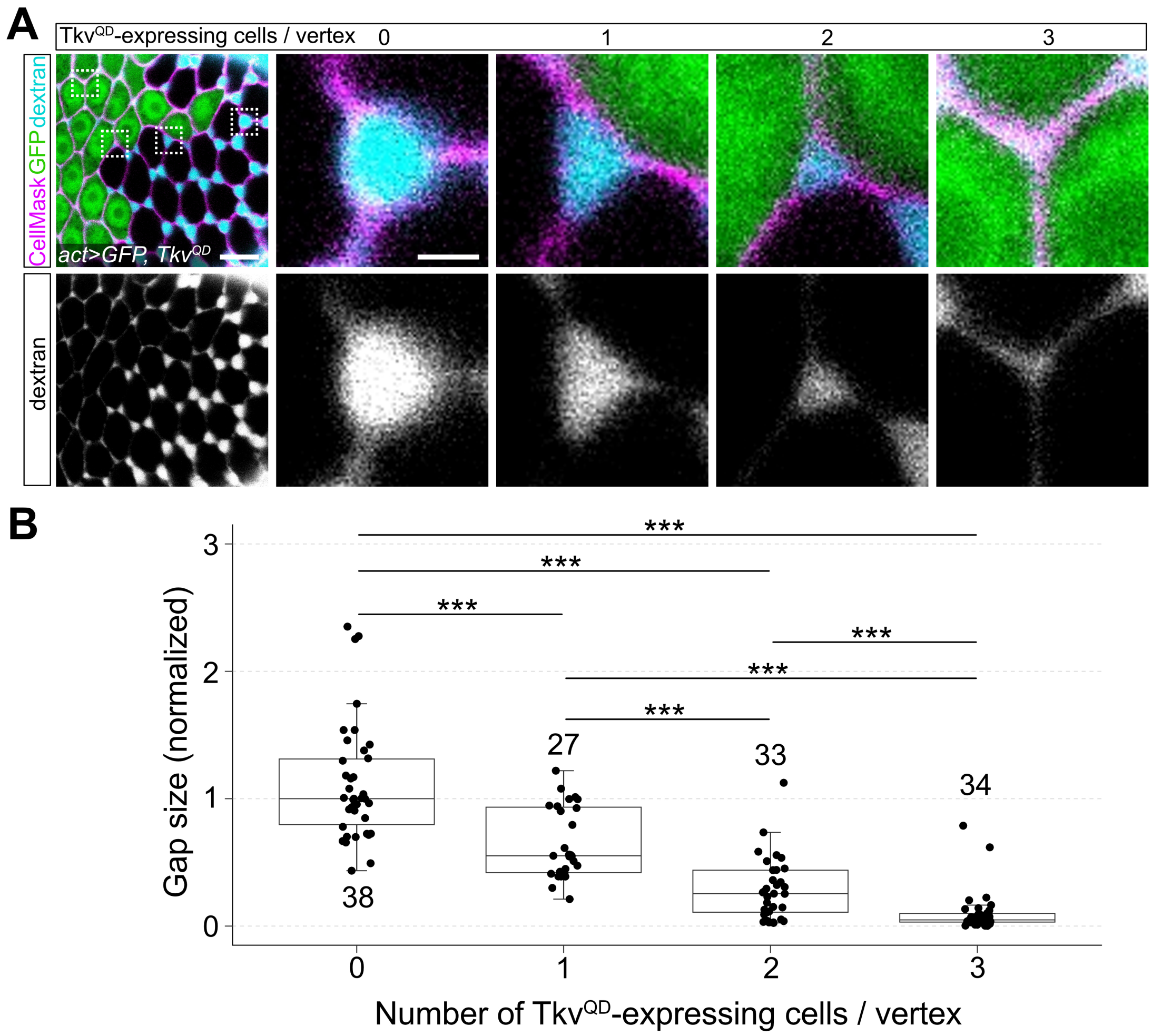

Fig. S1. Intercellular gap size scales with the number of Tkv^{QD}-expressing cells at mosaic vertices.

(A) Basolateral section of living follicle expressing Tkv^{QD} in GFP-marked cells (green). Dextran (cyan) labels intercellular gaps, CellMask (magenta) labels plasma membranes. Closeups show vertices containing the indicated numbers of cells expressing Tkv^{QD}. Single-channel images show dextran.

(B) Quantification of intercellular gap size (normalized) at vertices with the indicated number of Tkv^{QD}-expressing cells. p-values (Pairwise Wilcoxon Rank Sum Test): *** $p \leq 0.001$. The number of vertices analyzed for each class is indicated.

Scale bars: 10 μm; closeups 2.5 μm.

Journal of Cell Science • Supplementary information

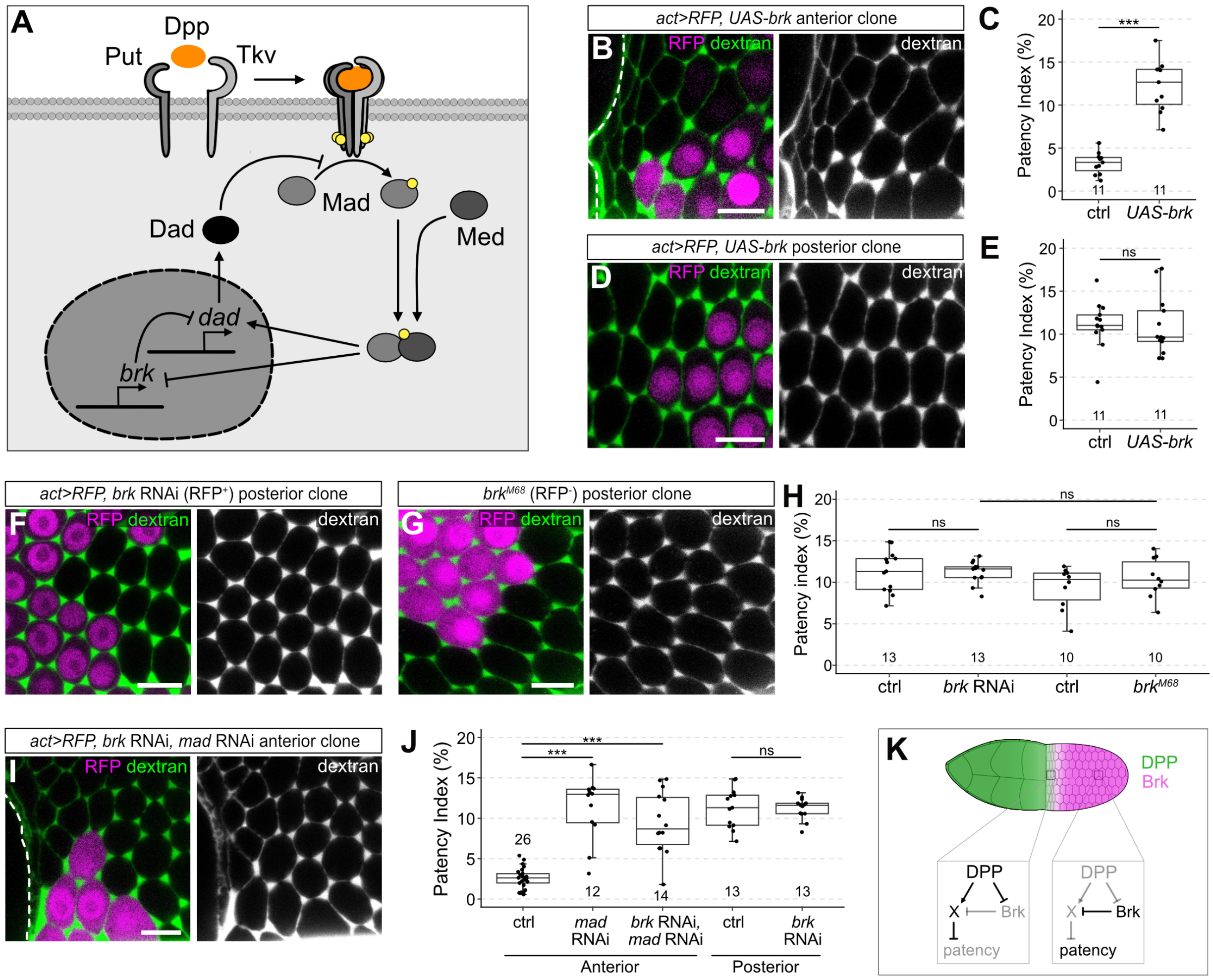

Fig. S2. The transcriptional repressor Brinker is not essential for patency.

(A) Scheme of TGF-β signaling pathway in *Drosophila melanogaster*. Dpp, Decapentaplegic (TGF-β ligand); Tkv, Thickveins (type-I Dpp receptor); Put, Punt (type-II Dpp receptor); Mad, Mothers against Dpp (SMAD); Med, Medea (co-SMAD); Dad, Daughters against Dpp (inhibitory SMAD); Brk, Brinker (transcriptional repressor). See text for details.

(B,D) Basolateral section of living follicle expressing *brk* in RFP-marked anterior (B) or posterior (D) follicle cell clones. Note the formation of ectopic intercellular gaps (dextran; green) upon *brk* misexpression in the anterior follicle cells. Dashed line marks the nurse cell-MBFC boundary.

(C,E) Quantification of patency index in the indicated genotypes. p-values (Pairwise Wilcoxon Rank Sum Test): *** $p \leq 0.001$; ns, not significant. Sample size (n) is indicated.

(F,G) Basolateral section of living follicles expressing *brk* dsRNA in RFP-marked clone (magenta; F) or lacking Brk in *brk*^{M68} null mutant clone (marked by absence of RFP; G) in

posterior follicle epithelium. Note that loss of Brinker does not affect the size of intercellular gaps (dextran; green) in the posterior follicle epithelium.

(H) Quantification of patency index in the indicated genotypes. p-values (Pairwise Wilcoxon Rank Sum Test): ns, not significant. Sample size (n) is indicated.

(I) Basolateral section of living follicle expressing *brk* and *mad* dsRNA in RFP-marked cells (magenta). Note that Brinker depletion does not suppress ectopic vertex opening (dextran; green) caused by *mad* depletion in anterior follicle epithelium. Dashed line marks the nurse cell-MBFC boundary.

(J) Quantification of patency index in the indicated genotypes. p-values (Pairwise Wilcoxon Rank Sum Test): *** $p \leq 0.001$; ns, not significant. Sample size (n) is indicated.

(K) Schematic model of the role of Brk-mediated transcriptional repression in regulation of patency in a stage 10A follicle. TGF-β signaling activity (green) and the transcriptional repressor Brk (magenta) form inverse gradients in the main body follicle epithelium. In anterior FCs, TGF-β signaling regulates expression of target genes (X) required to suppress patency both through activating mechanisms and through relief of Brk-mediated repression. Hence, ectopic expression of *brk* in anterior FCs cells can induce patency through repressing TGF-β target genes. In posterior FCs, TGF-β signaling is absent and Brk is expressed. Hence target TGF- β genes are not activated and are repressed by Brk. Neither overexpression nor knockdown of *brk* affect vertex opening, because TGF-β signaling is required for activation of the relevant target genes.

Scale bars: (B,D,F,G,I), 10 µm.

Journal of Cell Science • Supplementary information

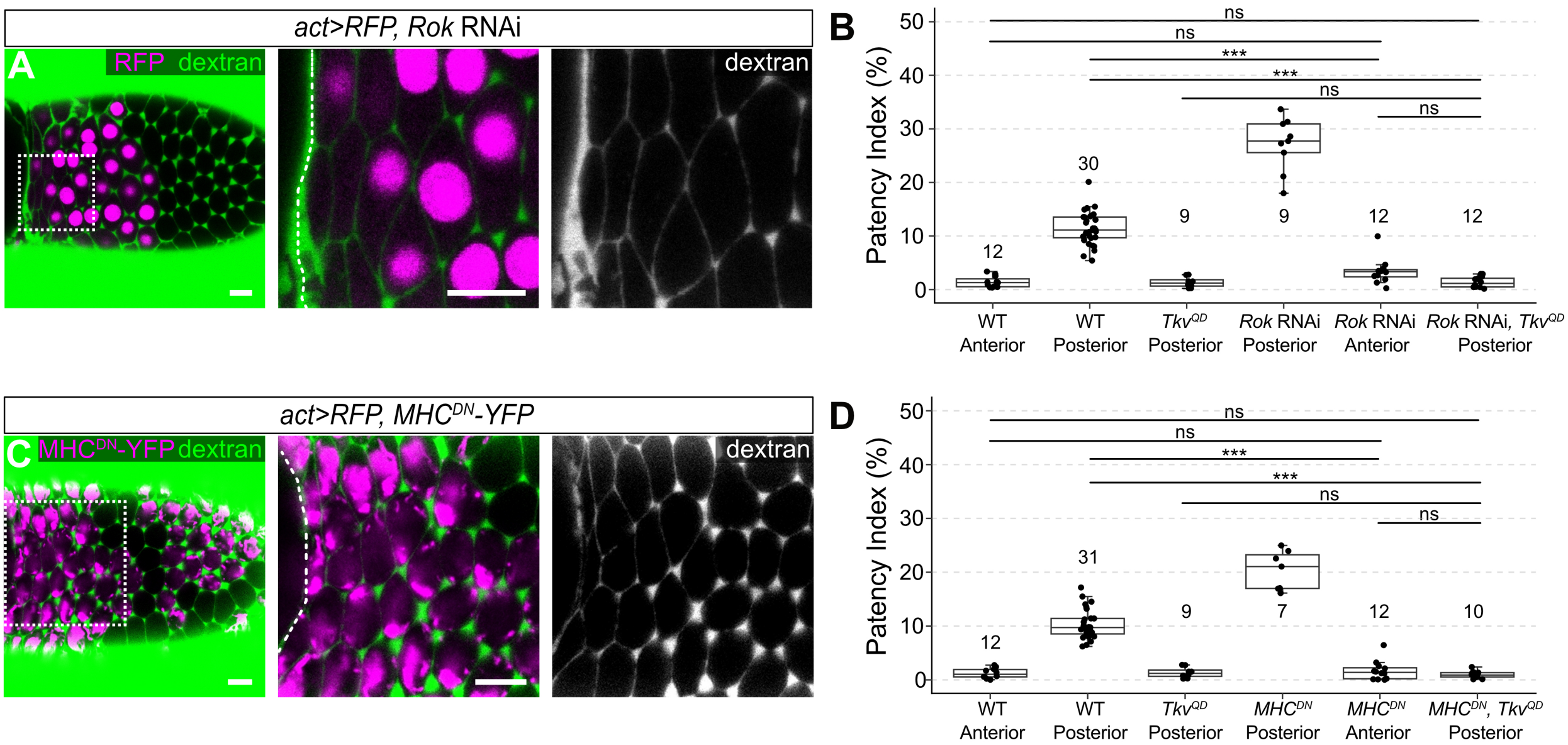

Fig. S3. Reducing MyoII activity is not sufficient to open vertices at physiological levels of TGF-β signaling.

(A,C) Basolateral section of living follicles expressing *Rok* dsRNA (A) or MHC^{DN}-YFP (C) in marked clones (magenta). Note that neither depletion of Rok (A) nor inactivation of MHC (C) leads to opening of vertices (dextran; green) in anterior FCs. Dashed lines in closeups mark the nurse cell-MBFC boundary.

(B,D) Quantification of patency index in the indicated genotypes. p-values (Pairwise Wilcoxon Rank Sum Test): *** $p \leq 0.001$; ns, not significant. Sample size (n) is indicated.

Scale bars: (A,C), 10 µm .

Journal of Cell Science • Supplementary information

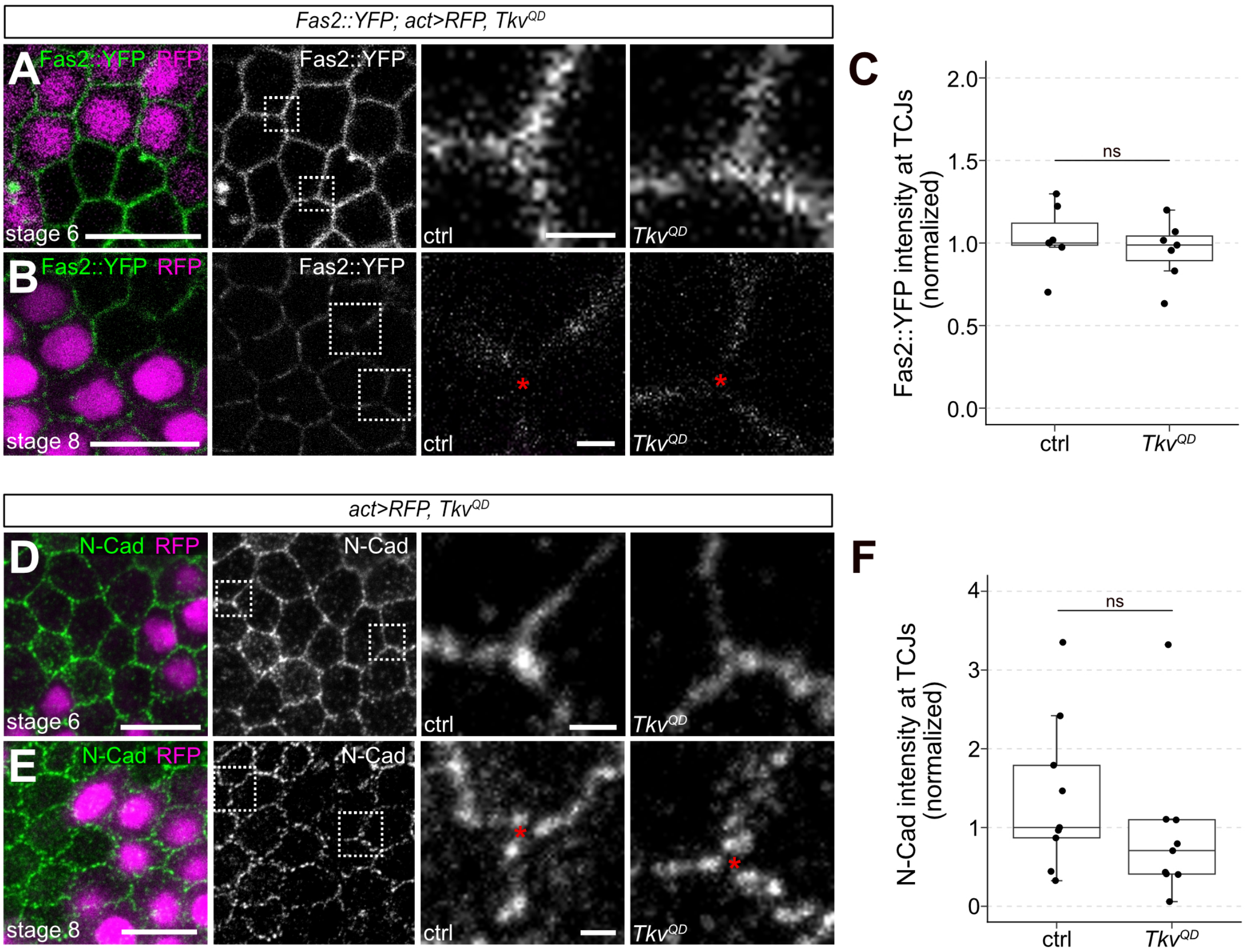

Fig. S4. Activation of TGF-β signaling does not stabilize Fas2 or N-Cad in follicle cells.

(A,B) Basolateral sections of living follicles expressing Fas2::YFP (green) ubiquitously and Tkv^{QD} in RFP-marked clones (magenta). Note that Fas2::YFP is present at vertices at stage 6 (A) in wild-type and Tkv^{QD}-expressing cells, whereas Fas2::YFP is removed from vertices (asterisks) and overall Fas2::YFP levels are reduced at stage 8 (B) in both genotypes.

(C) Quantification of Fas2::YFP at TCJs in control and Tkv^{QD}-expressing FC clones at stage 8 (n=7). p-values (Pairwise Wilcoxon Rank Sum Test): ns, not significant.

(D,E) Apical sections of fixed follicles expressing Tkv^{QD} in RFP-marked clones (magenta), stained for N-Cad (green). Note that N-Cad is present at vertices at stage 6 (C) in wild-type and Tkv^{QD}-expressing cells, whereas N-Cad is removed from vertices at stage 8 (D) in both genotypes.

(F) Quantification of N-Cad signals at TCJs in control and Tkv^{QD}-expressing FC clones at stage 8 (n=9). p-values (Pairwise Wilcoxon Rank Sum Test): ns, not significant.

Scale bars: (A,B,D,E), 10 µm, closeups 1 µm.

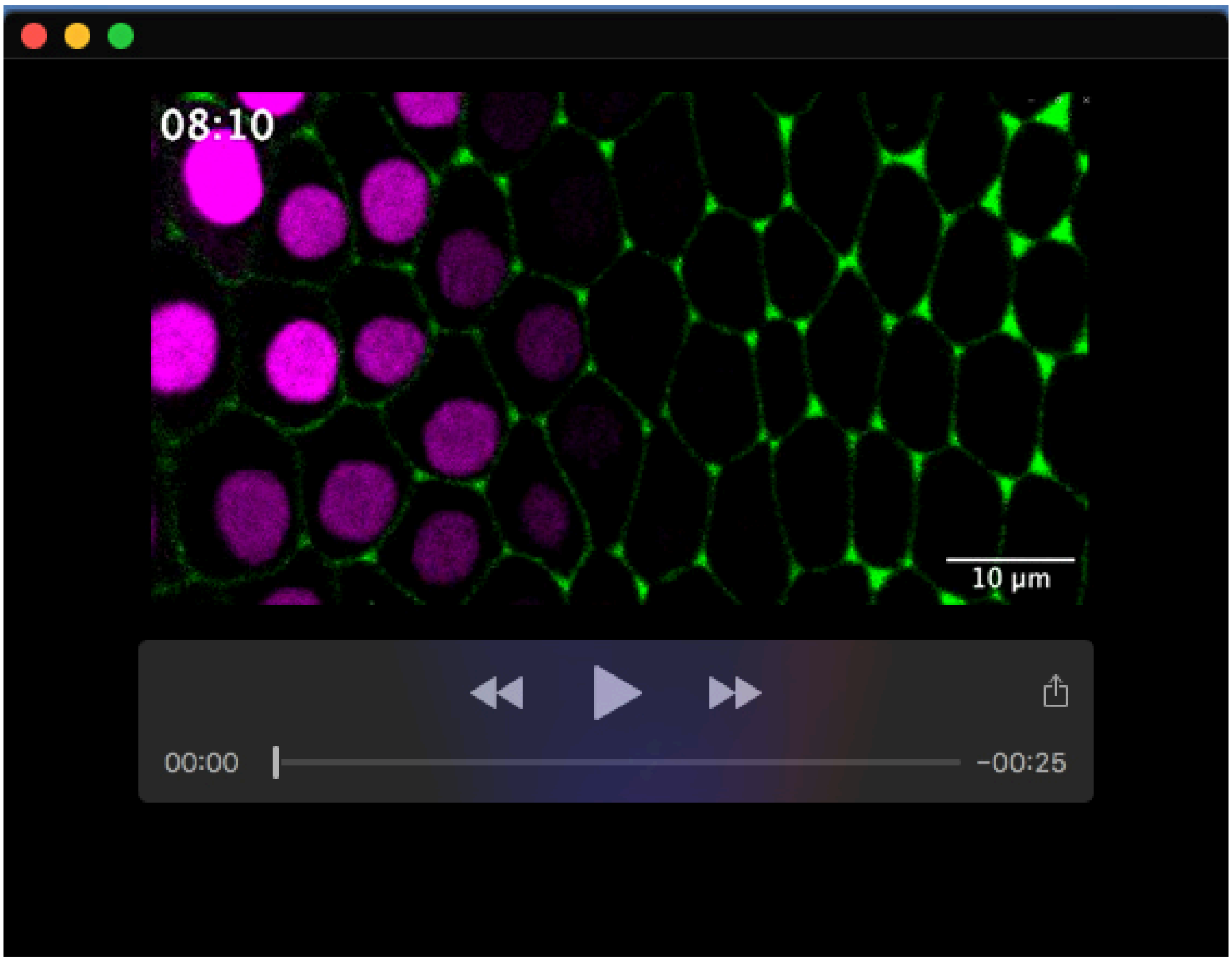

Movie 1. Anterior follicle cells resist to vertex opening induced by hypertonic treatment.

Time-lapse movie (basolateral section) of cultured stage 9 follicle expressing *dad*::GFP-nls (magenta), incubated in fluorescent dextran (green). A closeup of main-body follicle epithelium is shown. The follicle was exposed to hypertonic shock with DMSO (2.38% DMSO or 304 mosmol/L) at t=0 min. Note that addition of DMSO induces transient opening of vertices in posterior main-body FCs, but not in anterior FCs expressing *dad*::GFP-nls. Time (min) is indicated. Scale bar: 10 µm.

Journal of Cell Science • Supplementary information
