# Supplementary material for: TGF-β signaling modulates adhesion and actomyosin contractility to pattern epithelial permeability in Drosophila ovaries
Source: J Cell Sci. 2026 Oct 12;139(21):jcs264997. doi: 10.1242/jcs.264997 (PMC13741950; doi:10.1242/jcs.264997)
Supplement: Supplementary file 2 [file joces-139-264997_review_history.pdf]

TGF-β signaling modulates adhesion and actomyosin contractility to pattern epithelial permeability in *Drosophila* ovaries

Harshath Amal, Thea Jacobs, Max Lohrberg and Stefan Luschnig
DOI: 10.1242/jcs.264997

Editor: Richa Rikhy

Review timeline
Submission to Review Commons: 18 February 2026
Submission to Journal of Cell Science: 27 April 2026
Editorial decision: 8 May 2026
First revision received: 17 July 2026
Accepted: 7 August 2026

Reviewer 1

Evidence, reproducibility and clarity

In this manuscript, the authors explore how TGF ß signaling inhibits patency in the follicular epithelia of the Drosophila ovary. In this setting, patency is the opening of the tricellular junctions within the follicular epithelium (FE) covering the ovary to allow the transfer of yolk proteins into the underlying ovary. The authors first demonstrate that there is an inverse correlation between levels of Dpp signaling (based on a Dad-GFP reporter) to both the vertex (tricellular junction) opening size and the "circularity" of the FE cells, with Dpp signaling being highest at the anterior end. They show that activated Dpp signaling (Dad- GFP signal) is highest in the most anterior FE as are the highest levels of F-actin and MyoII (mCherry reporter) and that ectopic activation of Dpp signaling (using an activated receptor) in posterior FE cells is sufficient to induce higher levels of RhoI, junctional F-actin and MyoII at the tricellular junctions. However, neither knockdown of RhoI nor expression of a dominant negative form of MyoII have any impact on whether Dpp signaling blocks patency. Thus, although activated by Dpp signaling, MyoII activation is not required for Dpp to block patency. They show that Ecad is not present in the patent tricellular junctions, although it is present earlier and that Dpp signaling is required for enhanced levels of Ecad in anterior FEs and is sufficient to induce Ecad transcription (based on a lacZ reporter in the Ecad gene) and to increase Ecad protein levels. They show that Ecad is required to block patency regardless of Dpp signaling. They show that MyoII activity is not required for Dpp enhancement of Ecad protein levels. They show that Dpp signaling can increase p120cat levels and that p120ctn can increase Ecad levels. However, knockdown of P120cat has no effect on patency in either WT or TKV activated FEs.

The experiments are nicely down and illustrated, and the paper is well written.

I think the authors are overstating what they can conclude in both the title and abstract.

Significance

I think some of the conclusions cannot be made with the data in hand. Overall, the authors have shown that Dpp signaling enhances levels of several proteins that would be thought to block patency (Rho1, MyoII, F-Actin, p120cat, and Ecad (transcriptionally). They have shown that, except for Ecad, knockdown of most of these do not affect Dpp-dependent patency. However, showing that patency is severely enhanced in both WT and Dpp-activated cells with loss of Ecad is not sufficient evidence that Dpp signaling works through Ecad. Taking away Ecad is going to cause near or complete loss of AJs - thus, it is no surprise that patency is enormously increased

© 2026. Published by The Company of Biologists under the terms of the Creative Commons Attribution License (https://creativecommons.org/licenses/by/4.0/).

everywhere. Importantly, overexpression of Ecad (or of p120cat, which increases Ecad levels) did not block patency. Indeed, it seems like the only manipulation that mimics the effects of Dpp activation on patency is blocking endocytosis - so this seems a likely mechanism (it could also explain the higher levels of p120cat and/or Ecad at junctions). Overall, I agree that the authors can conclude that the Rho1 activation of MyoII observed downstream of Dpp signaling does not impact repression of patency. However, since overexpression of Ecad had no impact on patency, I think they can only conclude that the Ecad expression is enhanced downstream Dpp signaling but that this increase in Ecad expression is insufficient to block patency on its own. Thus, the the title and abstract should be modified to more accurately reflect the conclusions that can be made.

Minor suggestions

Figure 1G. Please clearly indicate where the clone of tkv8 null cells is located within the follicular epithelium.

In my opinion, both supplemental figures should be included in the main body of the paper. They make important points relevant to the conclusion. Figure S1 should be included as part of Figure 3. Figure S2 should be included a stand-alone figure, as there are currently only six figures in the manuscript and the panel in that figure showing that blocking endocytosis blocks patency is an interesting and potentially relevant finding.

In its current state, the paper is most appropriate for a specialized reader in the field of Drosophila oogenesis. If the authors were to follow up on a potential link between Dpp signaling and endocytosis and find such a link, then I think it would be of more general interest.

The time estimate below is based on not doing major experiments. If the authors were to follow up on the observation regarding endocytosis, it would be more in the 6 month range.

Reviewer 2

Evidence, reproducibility and clarity

Amal et al investigate how canonical TGF-β signaling regulates tricellular junction (TCJ) remodeling during follicular patency in the Drosophila ovarian follicular epithelium. Using genetic mosaics, quantitative imaging, and perturbations of signaling and cytoskeletal pathways, the authors show that TGF-β signaling suppresses patency in a cell-autonomous manner.

The authors convincingly show that TGF-β signaling prevents remodeling of tricellular junctions (TCJs) during patency. The figures and quantitative analyses are of an excellent standard, and I commend the authors on the clarity of their data presentation. Previous work from this laboratory demonstrated that patency is regulated by actomyosin activity. In the present study, the authors show that although TGF-β signaling increases actomyosin contractility, perturbation of downstream effectors of actomyosin contractility does not rescue the patency defect caused by constitutively active TGF-β signaling. This is a surprising and interesting result.

The authors then show that TGF-β regulates patency through effects on E-Cadherin. However, the mechanism by which TGF-β signaling regulates E-Cad remains somewhat unclear. Although the authors show that E-Cad levels appear elevated when TGF-β signaling is activated, E-Cad overexpression alone does not affect patency. The authors also test whether the effect reflects a broader change in adhesion proteins by examining Fas2 and N- Cad, which appear unchanged, suggesting that the effect is specific to E-Cad.

The introduction and discussion are scholarly and cite the appropriate literature.
Overall, the manuscript is rigorous, clearly presented, and ready for publication.

The experimental approaches are described in sufficient detail to allow reproduction, and the statistical analysis and quantification appear appropriate. The experiments appear adequately replicated, and the presentation of the quantitative data is clear.

Major comments:

© 2026. Published by The Company of Biologists under the terms of the Creative Commons Attribution License (https://creativecommons.org/licenses/by/4.0/).

N numbers for experiments Cells/Egg chambers appear to be missing. Please add these details.

Single images showing no change in the localization of Fas2 and NCad found in supplementary are not convincing. The authors should quantify this data.

Minor comments:

Figure 2A: Instead of sagittal sections through egg chambers, it may be more informative to show the imaging plane that highlights the surrounding follicular epithelium, which would better illustrate the spatial organization of the follicle cells.

Lines 73-85: Consider referring the reader to Figure 1A earlier in the text to help orient the reader to the architecture of the egg chamber.

It would also be helpful to include the abbreviation CPFC in the schematic in Figure 1A to make the terminology consistent with the text.

Significance

This is an exceptionally well-written and well-presented manuscript. The story presented is logical and the work is carefully executed with top-level figures and quantification. The manuscript is logically organized and controls and statistical tests are appropriate. The authors provide convincing evidence through careful genetic manipulations that TGF-ß signaling suppresses vertex opening primarily by reinforcing E-Cad-dependent adhesion rather than through actomyosin contractility.

A particular strength of the study is the clear dissection of two potential downstream pathways of TGF-ß signaling regulated patency- actomyosin contractility and E-Cad- mediated adhesion - and the demonstration that the suppression of patency depends primarily on E-Cad function. The manuscript represents a conceptual advance over the lab's previous work by demonstrating that patency is regulated by an upstream signaling pathway. Whereas earlier studies from this group established the cell biological mechanism of patency, this work shows that TGF-ß signaling acts as a regulatory input controlling this process.

The main limitation of the study is that the downstream molecular mechanism linking TGF-ß signaling to stabilization of E-Cad at tricellular vertices remains only partially defined. While the authors show that TGF-ß signaling increases E-Cad levels and promotes its retention at vertices many questions remain unclear as to how this is achieved. The data implicate p120- catenin as a possible contributor, but it does not appear to be required, leaving the mechanistic basis of E-Cad stabilization incompletely resolved.

The primary advance of the study is conceptual and mechanistic, showing that morphogen signaling can control TCJ integrity by stabilizing cadherin-based adhesion independently of actomyosin contractility. The work therefore advances our understanding of of how epithelial junction remodeling is regulated during development in the common model system of the Drosophila ovary.

In my opinion, the manuscript is exceptionally well presented and appropriate for publication essentially as-is.

The primary audience for this work will be researchers studying epithelial biology, morphogenesis and developmental cell biology, primarily those working in Drosophila. The manuscript will also be of interest to the broader cell and developmental biology community because it provides evidence for how signaling pathways and morphogen patterning regulates epithelial architecture and barrier function.

My expertise lies in epithelial morphogenesis, cell-cell adhesion, junction dynamics, and developmental cell biology and I use the Drosophila ovary as a model system. I reviewed the previous paper from this lab that went to Current Biology.

© 2026. Published by The Company of Biologists under the terms of the Creative Commons Attribution License (https://creativecommons.org/licenses/by/4.0/).

Reviewer 3

Evidence, reproducibility and clarity

The study by Amal et al. investigates how signaling cues regulate epithelial permeability using Drosophila oogenesis as a model system. During mid-oogenesis, a process known as patency occurs, in which tricellular junctions within the follicular epithelium transiently open, allowing yolk proteins to be transported from the hemolymph to the oocyte. The authors demonstrate that the spatial pattern of patency along the anterior-posterior axis of the egg chamber is inversely correlated with the activity gradient of TGF-ß signaling. They further show that TGF-ß signaling inhibits vertex opening and influences both actomyosin contractility and DE-cadherin levels. Importantly, although DE-cadherin is required for the TGF-ß-dependent suppression of vertex opening, elevated actomyosin contractility itself does not appear to be required for this effect.
Overall, this is a well-executed study that links a tissue patterning signal to the regulation of epithelial permeability. The experiments are clearly presented, and the quantification and statistical analyses are rigorous. I nevertheless have several points that should be addressed, either through additional experiments or through further discussion in the manuscript.

Main Points

1. Suppressing the effect of activated Tkv (TkvQD) by mad depletion is indeed good yet indirect evidence for the involvement of canonical (Mad-dependent) TGF-ß signaling. I believe a more direct way to reach this conclusion would be the generation of anterior mad loss of function clones which should mimic the tkv8 phenotypes.
2. On a more general note, most of the results of the paper are based on the hyperactivation of the pathway using TkvQD overexpression. I find this limiting for two reasons: First, the levels of TGF-ß signaling are abnormally high under these conditions. In this context, the interpretation of the contribution of TGF-ß induced MyoII and MyoII activity is unclear. The authors find that TGF-ß signaling activates MyoII activity, however inhibiting actomyosin contractility by various means did not restore vertex opening. This is however at levels of Tkv activity that are far beyond normal (TkvQD). At the same time, the same manipulations are sufficient to open vertices in cells that experience peak, endogenous levels of Tkv activity (anterior cells). Does endogenous Tkv signaling induce MyoII, MyoII activity, Rho1 in anterior levels? Addressing this in tkv8 mosaics would be helpful. I can imaging that, unlike Cadherin which seems to be epistatic to TkvQQ, it is a very difficult to exclude a contribution of TGF-ß mediated actomyosin contractility and there is probably not a good experiment to address this. However, I do not agree with the statement of line 174 "Although.... MyoII activity is dispensable for TGF-ß -mediated inhibition of vortex opening..." I think more appropriate would be to state that MyoII is dispensable for the abnormally/experimentally high TGF-ß signaling-mediated inhibition of vortex opening...". The explanation would be that under these conditions the exceptionally high TGF-ß signaling bypasses the need for MyoII (maybe through exceptionally high adhesion). This is apparently not the case at physiological levels of TGF-ß signaling at anterior cells.
Second, high levels of TkvQD, a protein that has been found to localize at junction in other systems, might have secondary effects in vertex opening for example by affecting their structural integrity or even by affecting endocytosis.
3. The effects of clonal manipulation of TGF-ß signaling within the clones are clear and solid. Although this would not affect the statements of this paper, it would be good if the authors could comment on the effects at clone boundaries. What happens to "hybrid" TCJ when wild- type cells (at the respective position and patency status) meet a clone with elevated or reduced TGF-ß signaling?
4. From a TGF-ß signaling-centric point of view: In this and other tissues, most of the TGF-ß signaling effects are mediated through the transcriptional repressor Brinker. The pattern of Brk expression is at the patency stage inverse to the pMad/ TGF-ß signaling activity (pMad represses brk transcription) and would in principle be identical in its graded profile with the pattern of vertex opening. Did the authors tried to manipulate levels of Brk? Is it possible to restore tkv8 phenotypes by simultaneously depleting brk?

Minor points

© 2026. Published by The Company of Biologists under the terms of the Creative Commons Attribution License (https://creativecommons.org/licenses/by/4.0/).

- Other than stated, not all egg chambers seem to be at stage 10 A in Fig. 1. Are the eggs shown in C older ?
- The box in 2A is very hard to see
- It is hard to correlate the dad::GFP-nls staining of 2A with the intensity profile of 2B. Is the quantification really at the sub-apical region as stated in the legend?

Significance

The findings of this study are highly significant and likely to be of broad interest, as they establish a strong link between a signaling pathway (TGF-ß signaling), best known for its role in gene expression and tissue patterning, and a highly dynamic cellular process-the remodeling of epithelial junctions that regulates epithelial permeability. While the involvement of TGF-ß signaling in this process is not entirely new (see Row et al., iScience, 2021), the present study provides a more detailed analysis and offers a molecular explanation linking TGF-ß signaling to epithelial junction patency.

Original submission

First decision letter

MS ID#: jcs.264997

MS TITLE: TGF-ß signaling modulates adhesion and actomyosin contractility to pattern epithelial permeability in Drosophila ovaries

AUTHORS: Harshath Amal; Thea Jacobs; Max Lohrberg; Stefan Luschnig

ARTICLE TYPE: Review Commons Transfer
Dear Dr Luschnig,

Many thanks for transferring your paper to Journal of Cell Science from Review Commons. I have reviewed the letter written with responses to the reviewers and the discussion on each of the points. Please revise the paper based on the comments of the reviewers and based on the suggestions for revisions made in your cover letter. We will then send the revised manuscript to the original reviewers for their comments on the revisions.

First revision

Author response to reviewers' comments

We thank the reviewers for their constructive and thoughtful comments on our manuscript. We appreciate that all three reviewers highlighted the quality of our work, as well as the significance and conceptual advance of our findings. We have revised the manuscript according to the reviewers' comments as detailed in our specific point-by-point responses below. Our responses and all changes in the revised text are highlighted in blue. With the new experimental data, analyses, and text revisions, we are confident that we address all points raised by the reviewers and that we have further strengthened the manuscript.

Summary of changes with respect to the original submission

Box plots:

Due to a setting in our analysis pipeline in R, the graphs in the original submission used box- plot

© 2026. Published by The Company of Biologists under the terms of the Creative Commons Attribution License (https://creativecommons.org/licenses/by/4.0/).

whiskers to indicate the standard deviation around the mean. In the revised manuscript, we modified all graphs so that the whiskers represent 1.5x the interquartile range, consistent with standard box-plot conventions.

Changes to figures:
Figure 1
Added images and quantification for *mad* RNAi anterior clones (panels J, L).
Marked the nurse cell-oocyte boundary in panel G.
Moved the scheme of TGF-ß signaling to revised Figure S2.

Figure 2
Removed the dotted line (line profile) in the close-up panel in Figure 2A.
Added images and quantification of pMyoII levels in anterior *mad* RNAi clones (panels H, I).

Figure 3
Rok epistasis data (Figure S1 of the original submission) was included.

Figure 4
No changes.

Figure 5
No changes.

Figure 6
Data on E-Cad endocytosis (Chc-DN, Rab5-DN, p120ctn) were moved here from original Figure S2. New data showing the effect of Rab5-DN was added to this Figure.

Figure 7
Is identical to Figure 6 in the original submission. No changes to the figure itself.

Figure S1 (new)
Shows new images and analyses of mosaic vertices.

Figure S2 (new)
Shows new data on the role of Brinker in patency. The scheme for TGF-ß signaling was moved here from the original Figure 1.

Figure S3 (new)
Shows new data (images and quantifications) for anterior *Rok* RNAi and MHC-DN-expressing clones.

Figure S4
Quantification was added for the effects of TGF-ß signaling on Fas2 and N-Cad (moved here from Figure S2 of the original submission).

Reviewer #1 (Evidence, reproducibility and clarity (Required)):

In this manuscript, the authors explore how TGF signaling inhibits patency in the follicular epithelia of the Drosophila ovary. In this setting, patency is the opening of the tricellular junctions within the follicular epithelium (FE) covering the ovary to allow the transfer of yolk proteins into the underlying ovary. The authors first demonstrate that there is an inverse correlation between levels of Dpp signaling (based on a Dad-GFP reporter) to both the vertex (tricellular junction) opening size and the "circularity" of the FE cells, with Dpp signaling being highest at the anterior end. They show that activated Dpp signaling (Dad-GFP signal) is highest in the most anterior FE as are the highest levels of F-actin and MyoII (mCherry reporter) and that ectopic activation of Dpp signaling (using an activated receptor) in posterior FE cells is sufficient to induce higher levels of RhoI, junctional F-actin and MyoII at the tricellular junctions. However, neither knockdown of RhoI nor expression of a dominant negative form of MyoII have any impact on whether Dpp signaling blocks patency. Thus, although activated by Dpp signaling, MyoII activation is not required for Dpp to block patency. They show that Ecad is not present in the patent tricellular junctions, although it is present earlier and that Dpp signaling is required for enhanced levels of Ecad in anterior FEs and is sufficient to induce

© 2026. Published by The Company of Biologists under the terms of the Creative Commons Attribution License (https://creativecommons.org/licenses/by/4.0/).

Ecad transcription (based on a lacZ reporter in the Ecad gene) and to increase Ecad protein levels. They show that Ecad is required to block patency regardless of Dpp signaling. They show that MyoII activity is not required for Dpp enhancement of Ecad protein levels. They show that Dpp signaling can increase p120cat levels and that p120ctn can increase Ecad levels. However, knockdown of P120cat has no effect on patency in either WT or TKV activated FEs.
The experiments are nicely down and illustrated, and the paper is well written.

We appreciate the reviewer's positive evaluation of our work, and we are grateful for the constructive and thoughtful comments.

I think the authors are overstating what they can conclude in both the title and abstract.

We acknowledge that the original title and abstract may read as too strong. We have revised both to more precisely capture what can be concluded from our results as detailed in our responses below.

Reviewer #1 (Significance (Required)):

I think some of the conclusions cannot be made with the data in hand. Overall, the authors have shown that Dpp signaling enhances levels of several proteins that would be thought to block patency (Rho1, MyoII, F-Actin, p120cat, and Ecad (transcriptionally). They have shown that, except for Ecad, knockdown of most of these do not affect Dpp-dependent patency. However, showing that patency is severely enhanced in both WT and Dpp-activated cells with loss of Ecad is not sufficient evidence that Dpp signaling works through Ecad. Taking away Ecad is going to cause near or complete loss of AJs - thus, it is no surprise that patency is enormously increased everywhere. Importantly, overexpression of Ecad (or of p120cat, which increases Ecad levels) did not block patency. Indeed, it seems like the only manipulation that mimics the effects of Dpp activation on patency is blocking endocytosis - so this seems a likely mechanism (it could also explain the higher levels of p120cat and/or Ecad at junctions). Overall, I agree that the authors can conclude that the Rho1 activation of MyoII observed downstream of Dpp signaling does not impact repression of patency. However, since overexpression of Ecad had no impact on patency, I think they can only conclude that the Ecad expression is enhanced downstream Dpp signaling but that this increase in Ecad expression is insufficient to block patency on its own. Thus, the the title and abstract should be modified to more accurately reflect the conclusions that can be made.
We agree with the reviewer´s point that TGF-ß signaling enhances E-Cad expression, but that this increase is insufficient on its own to block patency. This is a key result that was not stated clearly enough in the original submission. We have now clarified it by revising the title, abstract, and corresponding statements in the text accordingly.

Minor suggestions

Figure 1G. Please clearly indicate where the clone of tkv8 null cells is located within the follicular epithelium.

The tkv^8 clone is located in the region of the follicular epithelium overlying the nurse cell-oocyte boundary. To improve clarity, we added a dashed line marking this boundary in Fig. 1G and in similar panels, and noted its position in the figure legend.

In my opinion, both supplemental figures should be included in the main body of the paper. They make important points relevant to the conclusion. Figure S1 should be included as part of Figure 3. Figure S2 should be included a stand-alone figure, as there are currently only six figures in the manuscript and the panel in that figure showing that blocking endocytosis blocks patency is an interesting and potentially relevant finding.

We agree with the reviewer and followed the suggestions. We included the panels from original Figure S1 in the revised Figure 2. We included the data from original Figure S2 on E- Cad endocytosis (Chc-DN, Rab5-DN, p120ctn) in the revised Figure 6.
The remaining data from original Figure S2 (effects of TGF-ß signaling on Fas2 and N-Cad) are now shown in Supplementary Figure S4.

© 2026. Published by The Company of Biologists under the terms of the Creative Commons Attribution License (https://creativecommons.org/licenses/by/4.0/).

In its current state, the paper is most appropriate for a specialized reader in the field of Drosophila oogenesis. If the authors were to follow up on a potential link between Dpp signaling and endocytosis and find such a link, then I think it would be of more general interest.

We appreciate the comment. To test whether TGF-β signaling regulates E-Cad endocytosis, we used an antibody uptake assay in cultured follicles for visualizing E-Cad internalization directly. With this approach, we did not detect a significant change in E-Cad uptake upon manipulating TGF-β signaling in Tkv^{QD} clones. However, we cannot exclude a more spatially restricted effect, e.g., preferential modulation of E-Cad trafficking at cell vertices that may not be captured by this assay. In addition, we found that perturbing endocytic trafficking by expressing dominant-negative Rab5 (new panel Fig. 6D) or Rab11 (not shown) disrupts E- Cad removal from vertices and blocks patency, consistent with a requirement for membrane trafficking in vertex opening. Together, our findings support a key role for E-Cad trafficking in patency, while a direct link between TGF-β signaling and endocytosis remains an open question that would require significant future work beyond the scope of a revision.

While our experiments are conducted in the *Drosophila* follicular epithelium, we would like to note that the mechanism we identify—morphogen-dependent control of adhesion, actomyosin contractility and TCJ integrity—addresses a broader problem in epithelial biology: how spatially patterned signaling is translated into local regulation of barrier properties. By linking morphogen signaling to junctional remodeling and barrier function, we expect that our findings will be of broad interest to readers studying epithelial junction dynamics, tissue permeability, and the signaling programs that control barrier properties in epithelia.

The time estimate below is based on not doing major experiments. If the authors were to follow up on the observation regarding endocytosis, it would be more in the 6 month range.

Reviewer #2 (Evidence, reproducibility and clarity (Required)):

Amal et al investigate how canonical TGF-β signaling regulates tricellular junction (TCJ) remodeling during follicular patency in the Drosophila ovarian follicular epithelium. Using genetic mosaics, quantitative imaging, and perturbations of signaling and cytoskeletal pathways, the authors show that TGF-β signaling suppresses patency in a cell-autonomous manner.
The authors convincingly show that TGF-β signaling prevents remodeling of tricellular junctions (TCJs) during patency. The figures and quantitative analyses are of an excellent standard, and I commend the authors on the clarity of their data presentation. Previous work from this laboratory demonstrated that patency is regulated by actomyosin activity. In the present study, the authors show that although TGF-β signaling increases actomyosin contractility, perturbation of downstream effectors of actomyosin contractility does not rescue the patency defect caused by constitutively active TGF-β signaling. This is a surprising and interesting result.
The authors then show that TGF-β regulates patency through effects on E-Cadherin. However, the mechanism by which TGF-β signaling regulates E-Cad remains somewhat unclear. Although the authors show that E-Cad levels appear elevated when TGF-β signaling is activated, E-Cad overexpression alone does not affect patency. The authors also test whether the effect reflects a broader change in adhesion proteins by examining Fas2 and N-Cad, which appear unchanged, suggesting that the effect is specific to E-Cad.
The introduction and discussion are scholarly and cite the appropriate literature. Overall, the manuscript is rigorous, clearly presented, and ready for publication.
The experimental approaches are described in sufficient detail to allow reproduction, and the statistical analysis and quantification appear appropriate. The experiments appear adequately replicated, and the presentation of the quantitative data is clear.

We thank the reviewer for the positive evaluation of our work and for the constructive and thoughtful comments.

Major comments:

N numbers for experiments Cells/Egg chambers appear to be missing. Please add these details.

© 2026. Published by The Company of Biologists under the terms of the Creative Commons Attribution License (https://creativecommons.org/licenses/by/4.0/).

We apologize for the omission. We have now included the sample size (n) for each experiment in the respective graphs or figure legends.

Single images showing no change in the localization of Fas2 and NCad found in supplementary are not convincing. The authors should quantify this data.

We appreciate the comment. We included a quantification of the levels and localization of Fas2 and N-Cad in the revised manuscript (new Supplementary Figure S4).

Minor comments:

Figure 2A: Instead of sagittal sections through egg chambers, it may be more informative to show the imaging plane that highlights the surrounding follicular epithelium, which would better illustrate the spatial organization of the follicle cells.

Thank you for the suggestion. We chose to show a sagittal section because it most clearly reveals the myosin II gradient across the follicular epithelium. Following the reviewer's suggestion, we also examined a cortical section from the same specimen (see Figure 1 below). This view shows the spatial organization of the follicle cells, but the anterior-posterior gradient of apical myosin II is less apparent than in the sagittal section. For this reason, we kept the sagittal section in Fig. 2A. However, if requested, we will include it as an additional panel in the final version of the figure.

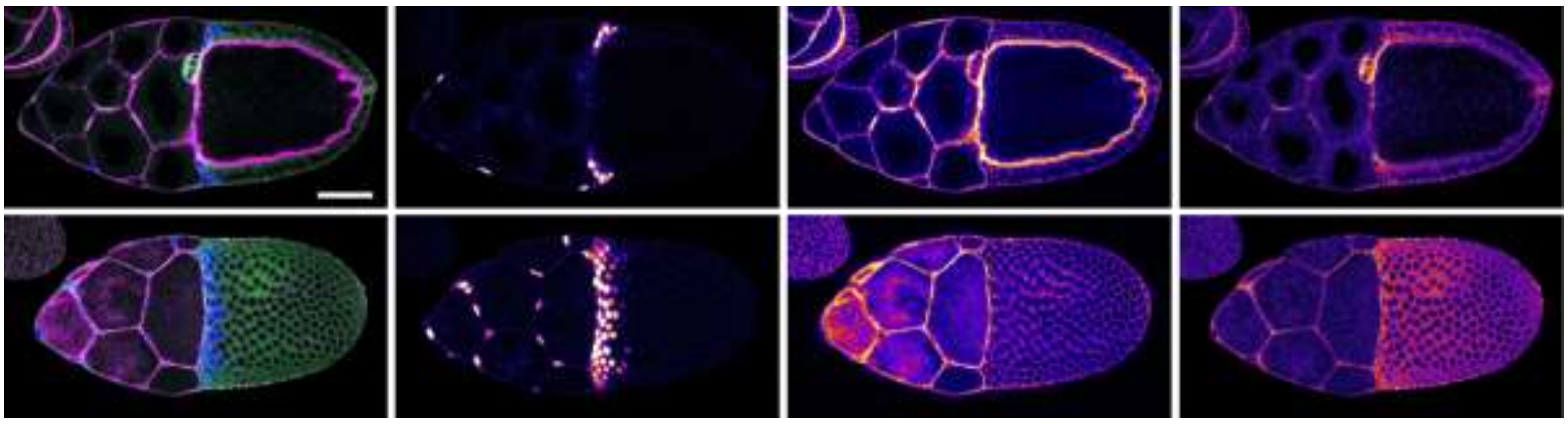

Figure 1: Mid-sagittal (top) and cortical (bottom) confocal sections of the same follicle (stage 10A) expressing *dad*::GFP-nls (blue in merge) and MyoII::mCherry (green in merge), stained with Phalloidin (magenta in merge). Intensities are color-coded using heatmap in single-channel images.

Lines 73-85: Consider referring the reader to Figure 1A earlier in the text to help orient the reader to the architecture of the egg chamber.

We now refer to Fig. 1A earlier in this section (line 71) to orient the reader to the architecture of the egg chamber before describing the specific features further down in the text.

It would also be helpful to include the abbreviation CPFC in the schematic in Figure 1A to make the terminology consistent with the text.

We have now included definitions of all relevant abbreviations, including "CPFC", in the schematic drawing in Figure 1A.

Reviewer #2 (Significance (Required)):

This is an exceptionally well-written and well-presented manuscript. The story presented is logical and the work is carefully executed with top-level figures and quantification. The manuscript is logically organized and controls and statistical tests are appropriate. The authors provide convincing evidence through careful genetic manipulations that TGF-β signaling suppresses vertex opening primarily by reinforcing E-Cad-dependent adhesion rather than through actomyosin contractility.
A particular strength of the study is the clear dissection of two potential downstream pathways of TGF-β signaling regulated patency- actomyosin contractility and E-Cad-mediated adhesion - and the

© 2026. Published by The Company of Biologists under the terms of the Creative Commons Attribution License (https://creativecommons.org/licenses/by/4.0/).

demonstration that the suppression of patency depends primarily on E-Cad function.
The manuscript represents a conceptual advance over the lab's previous work by demonstrating that patency is regulated by an upstream signaling pathway. Whereas earlier studies from this group established the cell biological mechanism of patency, this work shows that TGF-β signaling acts as a regulatory input controlling this process.
The main limitation of the study is that the downstream molecular mechanism linking TGF-β signaling to stabilization of E-Cad at tricellular vertices remains only partially defined. While the authors show that TGF-β signaling increases E-Cad levels and promotes its retention at vertices many questions remain unclear as to how this is achieved. The data implicate p120- catenin as a possible contributor, but it does not appear to be required, leaving the mechanistic basis of E-Cad stabilization incompletely resolved.

The primary advance of the study is conceptual and mechanistic, showing that morphogen signaling can control TCJ integrity by stabilizing cadherin-based adhesion independently of actomyosin contractility. The work therefore advances our understanding of of how epithelial junction remodeling is regulated during development in the common model system of the Drosophila ovary. In my opinion, the manuscript is exceptionally well presented and appropriate for publication essentially as-is.

We thank the reviewer for highlighting the conceptual advance of our findings. We show that TGF-β signaling increases E-Cad levels and promotes its retention at vertices, consistent with stabilization of cadherin-based adhesion at TCJs. We identify p120-catenin as a contributing effector; however, its nonessential role in our assays suggests that additional, potentially redundant mechanisms downstream of TGF-β likely cooperate to reinforce adhesion. Together, our findings support the central conclusion that TGF-β signaling regulates TCJ remodeling primarily by reinforcing cadherin-based adhesion rather than by altering actomyosin contractility, providing a mechanistic framework for how morphogen signaling controls epithelial junction remodeling during development.

The primary audience for this work will be researchers studying epithelial biology, morphogenesis and developmental cell biology, primarily those working in Drosophila. The manuscript will also be of interest to the broader cell and developmental biology community because it provides evidence for how signaling pathways and morphogen patterning regulates epithelial architecture and barrier function.
My expertise lies in epithelial morphogenesis, cell-cell adhesion, junction dynamics, and developmental cell biology and I use the Drosophila ovary as a model system. I reviewed the previous paper from this lab that went to Current Biology.

Reviewer #3 (Evidence, reproducibility and clarity (Required)):

The study by Amal et al. investigates how signaling cues regulate epithelial permeability using Drosophila oogenesis as a model system. During mid-oogenesis, a process known as patency occurs, in which tricellular junctions within the follicular epithelium transiently open, allowing yolk proteins to be transported from the hemolymph to the oocyte. The authors demonstrate that the spatial pattern of patency along the anterior-posterior axis of the egg chamber is inversely correlated with the activity gradient of TGF-β signaling. They further show that TGF- β signaling inhibits vertex opening and influences both actomyosin contractility and DE- cadherin levels. Importantly, although DE-cadherin is required for the TGF-β-dependent suppression of vertex opening, elevated actomyosin contractility itself does not appear to be required for this effect.
Overall, this is a well-executed study that links a tissue patterning signal to the regulation of epithelial permeability. The experiments are clearly presented, and the quantification and statistical analyses are rigorous. I nevertheless have several points that should be addressed, either through additional experiments or through further discussion in the manuscript.

We thank the reviewer for the positive evaluation of our work and for the constructive comments. We have addressed each point in detail below and revised the manuscript accordingly.

Main Points

1. Suppressing the effect of activated Tkv (TkvQD) by mad depletion is indeed good yet indirect

© 2026. Published by The Company of Biologists under the terms of the Creative Commons Attribution License (https://creativecommons.org/licenses/by/4.0/).

evidence for the involvement of canonical (Mad-dependent) TGF-ß signaling. I believe a more direct way to reach this conclusion would be the generation of anterior mad loss of function clones which should mimic the tkv8 phenotypes.

We appreciate the suggestion. To test the role of canonical TGF-β signaling more directly, we depleted Mad by RNAi in clones located in the anterior FE. These clones show open intercellular gaps (Fig. 1J,L) accompanied by decreased pMyoII levels (Fig. 2H,I) and reduced E-Cad enrichment (Fig. 4F,G), resembling the *tkv*^{8} loss-of-function condition (Fig. 1G,L; Fig. 4D,G). We included these new image data and corresponding quantifications in the revised manuscript to provide direct evidence that canonical, Mad-dependent TGF-β signaling suppresses vertex opening in the anterior FE.

2. On a more general note, most of the results of the paper are based on the hyperactivation of the pathway using TkvQD overexpression. I find this limiting for two reasons: **First, the levels of TGF-ß signaling are abnormally high under these conditions.** In this context, the interpretation of the contribution of TGF-ß induced MyoII and MyoII activity is unclear. The authors find that TGF-ß signaling activates MyoII activity, however inhibiting actomyosin contractility by various means did not restore vertex opening. This is however at levels of Tkv activity that are far beyond normal (TkvQD). At the same time, the same manipulations are sufficient to open vertices in cells that experience peak, endogenous levels of Tkv activity (anterior cells). **Does endogenous Tkv signaling induce MyoII, MyoII activity, Rho1 in anterior levels? Addressing this in tkv8 mosaics would be helpful.** I can imaging that, unlike Cadherin which seems to be epistatic to TkvQQ, it is a very difficult to exclude a contribution of TGF-ß mediated actomyosin contractility and there is probably not a good experiment to address this. However, I do not agree with the statement of line 174 "Although.... MyoII activity is dispensable for TGF-ß -mediated inhibition of vortex opening..." I think more appropriate would be to state that MyoII is dispensable for the abnormally/experimentally high TGF-ß signaling-mediated inhibition of vortex opening...". The explanation would be that under these conditions the exceptionally high TGF-ß signaling bypasses the need for MyoII (maybe through exceptionally high adhesion). This is apparently not the case at physiological levels of TGF-ß signaling at anterior cells.

This is an important point, and we think part of the reviewer´s concern may stem from a misunderstanding. We did not state that actomyosin manipulations are sufficient to open vertices in cells experiencing peak, endogenous levels of Tkv activity (i.e., anterior FCs). That said, we agree with the broader concern that results from experiments using pathway hyperactivation need to be interpreted with special caution. We therefore asked whether endogenous TGF-β signaling in anterior FCs correlates with increased MyoII levels, and whether MyoII is required to keep vertices closed also at physiological levels of TGF-β signaling.

First, we showed that MyoII (MyoII:mCherry) levels are elevated in anterior FCs, mirroring the endogenous TGF-β activity gradient (Fig. 2A,B), supporting the conclusion that physiological TGF-β signaling is associated with elevated contractility. Consistent with this, anterior *mad* RNAi clones show reduced pMyoII and E-Cad signals compared to neighboring wild-type cells (Fig. 2H,I; Fig. 4F,G), indicating that endogenous TGF-β signaling contributes to MyoII activation. We included these new data in the revised manuscript.

Second, we tested whether this increased contractility is required for maintaining vertices closed in anterior FCs (i.e., at peak levels of endogenous TGF-β activity). We found that suppressing MyoII activation in anterior FC clones by expressing Rok RNAi or Zip-DN did not induce vertex opening, despite reduced actomyosin contractility (Supplementary Figure S3). Thus, even at physiological levels of TGF-β signaling in anterior FCs, inhibiting actomyosin is not sufficient to allow vertex opening, suggesting that TGF-β signaling can bypass the requirement for actomyosin contractility, perhaps through elevated adhesion, as suggested by the reviewer. We included these new results in Supplementary Figure S3 and discuss their implications in the revised manuscript (line 203).

Second, high levels of TkvQD, a protein that has been found to localize at junction in other systems, might have secondary effects in vertex opening for example by affecting their structural integrity or even by affecting endocytosis.

© 2026. Published by The Company of Biologists under the terms of the Creative Commons Attribution License (https://creativecommons.org/licenses/by/4.0/).

We acknowledge that high-level expression of Tkv^{QD} could have secondary effects, *e.g.*, on junctional organization or endocytosis. We therefore revised the text to note that Tkv^{QD} represents an experimentally elevated signaling condition. We also clarified that our conclusions do not rely exclusively on constitutive signaling activation by Tkv^{QD}, as we also analyzed loss-of-function conditions (tkv^{8} and *mad* RNAi clones, as detailed above) that produce complementary phenotypes. We revised the text to make this logic more explicit and to note potential caveats of receptor hyperactivation (lines 204, line 292, line 320 in the revised manuscript).

3. The effects of clonal manipulation of TGF-ß signaling within the clones are clear and solid. Although this would not affect the statements of this paper, it would be good if the authors could comment on the effects at clone boundaries. What happens to "hybrid" TCJ when wild- type cells (at the respective position and patency status) meet a clone with elevated or reduced TGF-ß signaling?

We thank the reviewer for the excellent suggestion. Analyzing clone boundaries provides an informative way to assess the degree of cell-autonomy of TGF-ß activation on vertex opening. We therefore measured the size of individual vertex openings at vertices bordered by varying numbers (zero to three) of cells with activated TGF-ß signaling (Tkv^{QD}-expressing cells). Comparing these configurations allowed us to determine how strongly vertex opening depends on the signaling status of each participating cell, i.e. whether vertex opening scales with the number of TGF-ß-activated cells at the junction (suggesting a graded, cell-autonomous contribution) or whether the presence of a single TGF-ß-activated cell is sufficient to impose full vertex closure (suggesting a non-cell-autonomous effect). This analysis revealed that gap size decreased as the number of cells with activated TGF-ß signaling increased, indicating a strong cell-autonomous contribution to suppression of vertex opening. We included these new results and discussed their implications in the revised manuscript (Suppl. Fig. S1, line 142).

4. From a TGF-ß signaling-centric point of view: In this and other tissues, most of the TGF-ß signaling effects are mediated through the transcriptional repressor Brinker. The pattern of Brk expression is at the patency stage inverse to the pMad/ TGF-ß signaling activity (pMad represses brk transcription) and would in principle be identical in its graded profile with the pattern of vertex opening. Did the authors tried to manipulate levels of Brk? Is it possible to restore tkv8 phenotypes by simultaneously depleting brk?

We appreciate the suggestion. To test whether Brk contributes to the regulation of patency, we misexpressed Brk in anterior follicle cell clones. This induced ectopic vertex opening (Suppl. Fig. S2B,C), consistent with the notion that Brk represses TGF-ß-dependent transcription in this region. However, Brk overexpression in posterior clones did not further enhance patency (Suppl. Fig. S2D,E), and *brk* RNAi or brk^{M68} mutant clones in the posterior FE did not display detectable changes in patency (Suppl. Fig. S2F-H), indicating that absence of Brk is not sufficient to suppress vertex opening.

Furthermore, as suggested by the reviewer, we tested whether the effect of loss of upstream signaling can be rescued by simultaneously depleting Brk. Because generating the genotype to deplete Brk by RNAi in the background of tkv^{8} mutant clones was not possible within the revision timeframe, we instead generated clones in which *mad* and *brk* were simultaneously depleted by RNAi. This revealed that simultaneous depletion of *brk* did not suppress the ectopic anterior patency caused by *mad* RNAi (Fig. 1J,L; Fig. S2I,J), corroborating the notion that absence of Brk is not sufficient to suppress vertex opening.

We validated the efficiency of RNAi-mediated knockdown of *mad* and *brk* by examining clones homozygous for the respective loss-of-function mutants, which show comparable phenotypes (Suppl. Fig. S2F-H and data not shown for *mad*).

Together, these results argue against TGF-ß signaling acting solely through Brk as the limiting regulator of patency in the posterior FE and instead suggest that TGF-ß suppresses vertex opening via both de-repression of Brk target genes and Brk-independent gene activation mechanisms.

© 2026. Published by The Company of Biologists under the terms of the Creative Commons Attribution License (https://creativecommons.org/licenses/by/4.0/).

Similar context-dependent Brk-dependent and Brk-independent outputs of Dpp signaling have been reported in the embryo and larval imaginal discs (Marty et al., 2000, *Nat. Cell Biol.*). We included the new data in Supplementary Fig. S2 and in the text (line 150).

Minor points

- Other than stated, not all egg chambers seem to be at stage 10 A in Fig. 1. Are the eggs shown in C older ?

All egg chambers were staged using standard morphological criteria (follicle cell shape; onset and extent of centripetal cell ingression). The apparent differences between panels likely reflect differences in sample preparation and imaging conditions rather than developmental stage: panel (B) shows a fixed specimen, whereas panel (C) is live imaging, and different optical sections are shown (nuclei in (B) vs. FC membranes in (C)).

- The box in 2A is very hard to see

We increased the line thickness to improve visibility.

- It is hard to correlate the dad::GFP-nls staining of 2A with the intensity profile of 2B. Is the quantification really at the sub-apical region as stated in the legend?

We apologize for the confusion. This point was not clear in the original submission. The intensity profile for the dad::GFP-nls signals was taken along a line through the nuclei, whereas profiles for Phalloidin and MyoII::mCherry were taken in the sub-apical region of the FCs, as indicated in the merge panel of Fig. 2A in the original submission.
To clarify this point, the actual line profiles that were analyzed are shown in the version of Fig. 2A,B below. However, because the dashed lines obscure important features of the images, we have not included them in the final revised figure. Instead, we have revised the figure legend to explicitly state how signals in each channel were measured: "Normalized fluorescence intensities were measured along a line across the nuclei (*dad*::GFP-nls) or within the subapical region (Phalloidin, MyoII::mCherry)".

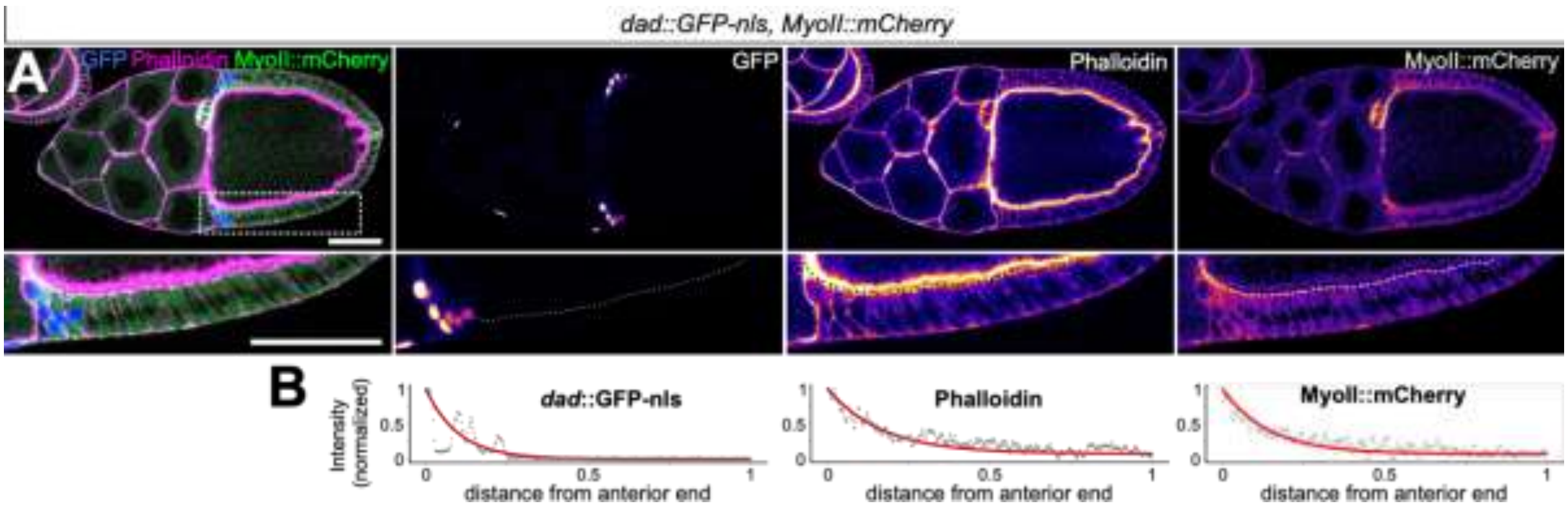

Reviewer #3 (Significance (Required)):

The findings of this study are highly significant and likely to be of broad interest, as they establish a strong link between a signaling pathway (TGF-ß signaling), best known for its role in gene expression and tissue patterning, and a highly dynamic cellular process-the remodeling of epithelial junctions that regulates epithelial permeability. While the involvement of TGF-ß signaling in this process is not entirely new (see Row et al., iScience, 2021), the present study provides a more detailed analysis and offers a molecular explanation linking TGF-ß signaling to epithelial junction patency.

We thank the reviewer for the enthusiastic and thoughtful evaluation and for highlighting the conceptual advance of our findings. We appreciate the recognition that our study provides a mechanistic framework connecting TGF-ß signaling to junctional remodeling and epithelial

© 2026. Published by The Company of Biologists under the terms of the Creative Commons Attribution License (https://creativecommons.org/licenses/by/4.0/).

permeability.

Second decision letter

MS ID#: jcs.264997R1

MS Title: TGF-ß signaling modulates adhesion and actomyosin contractility to pattern epithelial permeability in Drosophila ovaries

Authors: Harshath Amal; Thea Jacobs; Max Lohrberg; Stefan Luschnig

Article Type: Review Commons Transfer

Dear Dr Luschnig,

I am happy to tell you that your manuscript has been accepted for publication in Journal of Cell Science, pending standard publication integrity checks.

Thank you for sending your manuscript to Journal of Cell Science through Review Commons.

Reviewer 1

SUMMARY OF THE ADVANCE MADE IN THIS PAPER AND ITS POTENTIAL SIGNIFICANCE TO THE FIELD

As previously stated, the primary advance of the study is conceptual and mechanistic, showing that morphogen signaling can control TCJ integrity by stabilizing cadherin-based adhesion independently of actomyosin contractility. The work therefore advances our understanding of of how epithelial junction remodeling is regulated during development in the common model system of the Drosophila ovary.

SUGGESTIONS TO AUTHORS

The authors have addressed all of the comments of the reviewers to a high standard and I recommend this manuscript for publication.

Reviewer 2

SUMMARY OF THE ADVANCE MADE IN THIS PAPER AND ITS POTENTIAL SIGNIFICANCE TO THE FIELD

The authors show that TGFbeta signaling inhibits patency of tri-cellular junctions of the follicular epithelia by transcriptionally upregulating ECadherin expression and preventing ECadherin endocytosis, revealing how localized signaling pathways are linked to functional changes in epithelial permeabilty to build a better oocyte (where the localized transport of yolk proteins into the primary oocyte is maximized). Surprisingly, the authors also show that the TGF-beta dependent upregulation of MyoII activity through Rho/Rok that they also observed does not contribute to decreases in patency. Although the authors provide evidence that upregulation of p120-catenin can stabilize membrane E-Cad, p120-catenin is not required to block patency. Thus, the exact moleculr link between TGF-beta signaling and Ecad endocytosis remains unknown. Nonetheless, the findings in the current version of the manuscript are important and advance our understanding of this interesting biological process.

SUGGESTIONS TO AUTHORS

Thank you for addressing the concerns of all three reviewers - the manuscript is much stronger with the corresponding changes (experimental and textual) that were made. As far as I am concerned, no further revision is required.

© 2026. Published by The Company of Biologists under the terms of the Creative Commons Attribution License (https://creativecommons.org/licenses/by/4.0/).

Reviewer 3

The authors addressed and clarified all my concerns (and I think the concerns and questions of the other 2 reviewers) with new experiments and data in this carefully crafted revised version. I congratulate the authors on their interesting and solid story!

© 2026. Published by The Company of Biologists under the terms of the Creative Commons Attribution License (https://creativecommons.org/licenses/by/4.0/).
